# The Role of Radiotherapy in Hodgkin's Lymphoma: What Has Been Achieved during the Last 50 Years?

**DOI:** 10.1155/2015/485071

**Published:** 2015-02-01

**Authors:** Magdalena Witkowska, Agata Majchrzak, Piotr Smolewski

**Affiliations:** Department of Experimental Hematology, Medical University of Lodz, Copernicus Memorial Hospital, Ciolkowskiego 2, 93-510 Lodz, Poland

## Abstract

Currently, Hodgkin's lymphoma (HL) has an excellent clinical outcome, with overall survival of approximately 90% in early stages of the disease. Based on young age of the majority of patients at the time of diagnosis and their long survival time, increased attention has been focused on long-term toxicity of therapy. While novel, directly targeting antitumor agents, with an excellent safety profile, have been developed for HL treatment, the role of radiotherapy is still debated. Radiotherapy may induce cardiovascular disease and impairment of thyroid or pulmonary function and, most importantly, may lead to development of secondary cancers. As a consequence, the current radiation therapy planning paradigm is mainly focused on a reduction of field size. As it was investigated in clinical trials regional therapy is as effective as extended field radiotherapy, but less toxic. Although chemotherapy is the mainstay of HL treatment, consolidative involved field radiation therapy is still considered to be the standard of care in both early and advanced stages. Recently, further field reduction has been investigated to further decrease the late radiation-induced toxicity. In this paper we describe the role and safety profile of radiotherapy in the past and present and hope for the novel techniques in the future.

## 1. Introduction

Hodgkin's lymphoma (HL) is a clonal malignancy of the lymphatic system that arises from B-cells of germinal and postgerminal centres. The frequency of HL is around 10% of all lymphoma types and approximately 0.6% of malignant diseases in Western European countries [1]. The disease occurrence in adults shows two peaks: the first is observed in young adulthood (age ranged from 15 to 30 years old) and the second in group over 55 years old [2].

Based on differences in the histological picture and the neoplastic cell phenotype HL can be divided into two distinct subgroups: classical HL (cHL) which is recognized in majority of patients (95%) and nodular lymphocyte-predominant HL (5%). cHL type can be further divided into four subtypes: lymphocyte-rich lymphocyte-predominant (LR-LP), nodular sclerosis (NS), mixed cellularity (MC), and lymphocyte-depleted (LD) [3]. Typical for all subtypes of cHL is the presence of neoplastic Reed-Sternberg (RS) cells, which are not observed in any other neoplastic diseases. Tumor is comprised of RS cells in minority, while the majority is an inflammatory background, crucial for growth and survival of cancer cells [4]. Microenvironment is composed of various cell types including lymphocytes, eosinophils, histiocytes, and plasma cells, which interact with numerous cells including CD4+ and CD8+ T cells, B lymphocytes, plasma cells, or dendritic cells, through secretion of different cytokines and chemokines. The complex microenvironment interactions are unique among lymphomas and are responsible for initiation and progression of HL.

For a long time, before finding a reliable treatment, HL was a fatal disease with progressive presentation and poor clinical outcome. Nowadays, it can be successfully treated with chemo- and radiotherapy (RT) in great majority of patients, with long-term survival exceeding 80% [5]. Still there are approximately 30% of patients who relapse after first line therapy [6]. For all transplant eligible patients, salvage chemotherapy with consolidative autologous stem cell transplantation (autoSCT) is a standard of care. Unfortunately, prognosis for those groups is rather poor with possibility to achieve a complete remission (CR) in less than 50% with a median overall survival (OS) of approximately 2 years [7].

It is widely accepted that HL is extremely sensitive to radiation therapy. In early favorable disease involved field RT (IF-RT) with 20 Grey (Gy) in combination with 2 cycles of first line chemotherapy composed of adriamycin, bleomycin, vinblastine, and dacarbazine (ABVD regimen) is a gold standard of treatment with observed long-term disease control [8]. What is important, the dose of radiation required to treat HL is significantly lower than in solid tumors. Still, acute and long-term toxicity, including secondary malignancies, as well as heart and lung diseases, occurs after radiation exposure and remains a main concern.

While recently a great number of novel, directly targeted agents with an excellent safety profile have been developed for HL treatment, the role of RT is debated. As a consequence, the current radiation therapy planning paradigm is mainly focused on a reduction of size of radiated fields. So far it was discovered that regional therapy is as effective as extended field RT (EF-RT), whereas less toxic.

## 2. History of Hodgkin's Lymphoma Treatment

It is believed that the history of HL starts in 1832 with the discovery of abnormalities in the lymph nodes, first described by English pathologist Sir Thomas Hodgkin and named after him, although the earliest reference to the condition was probably provided by Malpighi in 1666 [9]. In early years of the next century, HL was differentiated from other types of lymphomas with similar clinical manifestation, mainly due to its typical morphologic presentation.

The attempt to treat HL began just after the discovery of X-rays at the beginning of 20th century, when the dramatic regression of enlarged lymph nodes was observed. In work published by Pusey it was described that patients both with HL and sarcoma could be successfully cured with exposure to X-rays [10]. Unfortunately, after impressive preliminary report, responses were still only partial or did not last long enough. Later, Gilbert established the concept of extending the radiation fields into the adjacent clinically uninvolved areas [11]. In 1940s further technological development in higher radiation dose, safer profile, and better X-ray penetrating machines were built.

Another breakthrough in HL treatment was observed in 1950s with the development of nitrogen mustard. In 1946 Goodman and Wintrobe from the Yale University discovered that HL is not only radiosensitive but also chemosensitive cancer [12]. It is believed that this work could be the first phase I/II clinical trial on record. Later, as a consequence, novel drugs and their combinations were widely investigated in HL patients. Few years later in 1947 Alpert and Peterson published results proving that nitrogen mustard causes dissolution of tumor masses in patients with HL [13].

Another important step was introduction of combined chemotherapy composed of nitrogen mustard, vincristine, prednisone, and procarbazine (MOPP regimen) in 1970 by Devita Jr. et al. [14]. It allowed for the first time to cure patients, even with advanced clinical stages (IIIB and IV according to Ann Arbor classification). It was a revolution, which after 6 to 8 cycles of MOPP could provide CR at 60% to even 80% and the 10-year survival rate could be reached in more than half of patients. Further improvement observed during the period 1960–1990 was the most spectacular in treatment of all known malignant diseases. Between 1974 and 1982 in the Milan Cancer Institute, Italy, effectiveness of ABVD and MOPP was compared in the prospective, randomized trial. It was the first step that lead to introduction of ABVD into HL therapy, and this regimen finally becomes standard first line treatment till today [15].

At the same time RT techniques were evaluated toward higher effectiveness and less toxicity. In 1950 Petres [16] published unrandomized results showing CR after treatment with RT only in patients with HL. Next, due to improved X-ray penetration and adapted involved areas it became possible to cure HL patients, especially in limited stage of disease. In 1962 Kaplan published data on EF-RT in patients with localized disease [17]. In this work 5-year survival for limited stage was approximately 70% [17]. Although high volume RT occurred to be related with delayed toxicity involving secondary cancers, heart and lung disorder, or endocrine dysfunction, it was useful for further treatment development. In randomized clinical trials evaluating EF-RT versus IF-RT although progression free survival (PFS) after EF-RT was longer, the overall survival (OS) was similar for both RT methods in early-stage HL [18].

In order to cure more patients, especially with advanced stage, programs combined RT and chemotherapy were developed. Although the response rates were significantly better, the number of complications both during treatment and long term was much higher. Nowadays, RT along with ABVD regimen is still standard of care in early stages of the disease, as well as in advanced stages, when there is a large residual mass observed after chemotherapy according to European Society for Medical Oncology (ESMO) guidelines [19].

## 3. Toxicity of Radiotherapy

As was already mentioned, for many years, standard RT treatment for HL patients was EF-RT, used for delivering radiation to large areas of the body. EF-RT involves the irradiation of not only affected lymph node regions, but also adjacent nodal regions which may soon get affected [20]. Although the overall 10-year PFS was approximately 80% for limited stage of the disease, prolonged follow-up of the patients reveals the late toxicity of such approach [21]. Currently, due to complications this method is completely replaced with much less harmful IF-RT.

Toxicity connected with RT can be divided into early and long-term side effects. They depend on dose of X-rays and exact place, where the radiation is aimed. The most common acute complications are connected with skin changes similar to sunburn, which slowly fades away. Other possible short-term side effects include fatigue, dry mouth, change of taste, nausea, or diarrhea. If RT is administered to several areas, or briefly after chemotherapy, impairment of bone marrow function can be observed including anemia, low platelets count, and decreased level of white blood cells in peripheral blood.

According to different clinical studies HL survivors are also exposed to more severe long-term treatment-related morbidity (TRM). The most serious is the development of secondary cancer in the part of the body that was exposed to radiation; others include deteriorate cardiovascular, pulmonary, and thyroid function. As a result it leads to substantial morbidity and the quality of life can be significantly affected among radiated patients [22].

It has been already observed in literature that after the irradiation of the neck region approximately half of the patients will suffer from hypothyroidism and 20% will develop thyroid nodules [23]. It can be detected by elevated level of thyroid-stimulating hormone (TSH). In this case thyroid supplementation is recommended even if no symptoms are detected in order to prevent hypothyroidism. Cardiovascular disease that may occur after RT includes coronary artery disease, myocardial injury, valvular disease, or pericardial fibrosis [24]. In cardiac complications radiation dose is extremely important. It was observed that risk of congestive cardiac failure, pericardial disease, and valve abnormalities is more likely in patients exposed to more than 15 Gy [25]. Furthermore, according to Mulrooney et al. the risk of myocardial ischemia is increased with higher radiation doses, with an overall hazard ratio (HR) of more than 12 for those treated with mediastinal radiotherapy in childhood [26].

Last but not least, HL survivors will suffer from secondary malignancies, with the most common breast cancer in female and lung cancer in male patients [27]. So far, it was observed in several trials that women treated with RT for HL have strongly elevated risk of developing breast cancer compared with the general population [28]. The risk is inversely related to age at HL diagnosis and is the highest for women who are exposed to RT around puberty period and decreases progressively for the older [29]. In the male group of HL patients lung cancer was the most common secondary neoplasm. The incidence was significantly increased compared to control group, with the interval between diagnosis and cancer development of lung cancer varying from 2 to even 24 years in one case [30]. What is more, increased risk of myelodysplasia and acute myeloid leukemia may be observed after EF-RT, but the exact analyses of RT complications are difficult due to simultaneous treatment with alkylating agents, which is connected with significant risk as well [31]. As far as second malignancies are concerned, the cumulative incidence of HL patients is from 10 to 13% at 15 years of observation and this risk increases every year [32].

In order to reduce the risk of RT-related toxicity, the exact dose of radiation needed is carefully calculated and the main focus is to irradiate involved lymph node as accurately as it is possible. Shields may also be placed over nearby parts of the body to protect them from the radiation. In girls and young women, the ovaries may be moved out of the way with minor surgery before radiation is given to help preserve fertility.

## 4. Attempts to Omit Radiotherapy

After the success of chemotherapy and upcoming RT-induced toxicity there was a hypothesis to make an attempt to avoid RT at low risk HL patients. The results of smaller, single-center studies suggested that 6 cycles of ABVD were effective enough for early stage patients. In a study by Canellos et al. in 71 investigated HL patients with early favorable stage of the disease, there were only 6 recurrences observed among all investigated subjects [33]. Moreover, after 5 years of follow-up no deaths were recorded.

Similar study (HD6 trial) was designed by Eastern Cooperative Oncology Group (ECOG) where in stage IA or IIA nonbulky HL patients ABVD therapy alone was compared with subtotal nodal radiation with or without chemotherapy [34]. It was observed that patients treated with ABVD-only group had significantly higher OS than the radiation therapy group. In the RT group the mortality was higher mainly due to late treatment complications such as second cancers and cardiac events. Although the HD6 trial suggests that ABVD alone can be a therapeutic option for stage IA or IIA nonbulky HL population, this strategy is still controversial and not confirmed by other large clinical studies.

**Figure 1:**
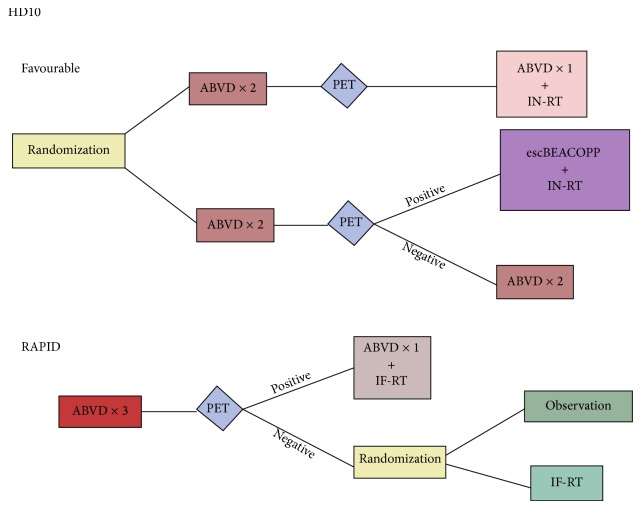
Schema for German HD10 and English RAPID Trial. ABVD: adriamycin, bleomycin, vinblastine, and dacarbazine, PET: positron emission tomography, IN-RT: involved nodal radiation therapy, IF-RT: involved field radiation therapy, escBEACOPP: bleomycin, etoposide, doxorubicin, cyclophosphamide, vincristine, procarbazine, and prednisone.

This tendency was not confirmed by larger multicenter trials. Both National Cancer Research Institute (NCRI) RAPID trial and European Organisation for Research and Treatment of Cancer (EORTC) H10 studies were planned in order to compare chemotherapy alone with a treatment composed of ABVD with consolidative RT [34, 35]. RAPID study was designed to reduce amount of chemotherapy as well as limit or even avoid RT. In 602 early stage HL patients positron emission tomography (PET) was performed after three ABVD cycles. Patients with negative interim PET were further randomized into two arms: IF-RT (209 patients) or observation arm (211 patients). Group with positive interim PET received both one ABVD cycle and IF-RT. After 3-year observation PFS and OS were 85.9% and 93.9% respectively, for PET positive patients. PET negative patients, had 3-year PFS 90.7%, for group randomized to observation, while group randomized to IF-RT had 3-year PFS 97% (*P* = 0.03). The second large study by German Hodgkin's Lymphoma Study Group (GHSG) evaluated 1370 newly diagnosed patients with early stage HL. Patients were randomized to one of four groups: 4 cycles of ABVD with 20 Gy IF-RT, 4 cycles of ABVD with 30 Gy IF-RT, 2 cycles of ABVD with 20 Gy IF-RT, or 2 cycles of ABVD with 30 Gy IF-RT. The results show advantage in clinical response of patients who received 30 Gy as compared to 20 Gy irradiation. In both studies 4% to 6% improvement in both one- and two-year PFS was observed in favor of combined modality treatment, even in the most favorable interim PET negative group (both schema depicted in Figure 1).

The role of RT in patients with advanced stage HL was evaluated in HD15 trial. This was large, prospective, randomized clinical trial conducted by GHSG group. According to this paper PET-guided RT after six cycles of BEACOPP (escalated) was much more effective and less toxic than eight cycles of the same chemotherapy regimen [36]. The negative predictive value for PET at 12 months was 94.1% and only 11% of investigated patients received additional RT.

The situation is different as far as children with HL diagnosis are concerned. It has been already proven that RT in younger patients can induce not only secondary malignancy and cardiovascular disease, but also the effects upon skeletal growth and maturation. There were a randomized CCG 5942 trial by North American Children's Oncology Group that examined chemotherapy alone approach. Children who achieved CR after chemotherapy were randomized into two groups: low-dose (21 Gy) IF-RT or no further treatment [37]. In a group of 498 patients after a median 7.7 years of observation there was a significant difference in event-free survival favoring the radiotherapy group (93% versus 83%, *P* = 0.004). What was interesting approximately is that 90% of recurrences were in the initial disease site, which would have been irradiated in the other trial arm. On the other hand there were no difference in OS, with 10-year estimated survival rates of 97% and 96%.

There is also a question, whether addition of RT to high-dose chemotherapy and autoSCT can improve the outcomes of HL patients with relapsed and refractory disease. In the study by Kahn et al. 92 patients were analyzed in a case-control design [38]. Group of 46 patients who received IF-RT within 2 months of SCT were compared to 46 patients who did not receive IF-RT. The use of RT was associated with better disease control and less progression observed in sites of prior disease involvement.

So far, according to data presented above, chemotherapy alone should not be considered as a treatment strategy that incorporates RT in all cases. As a result 20 Gy of IF-RT remains standard treatment for patients with stages IA and IIA, favorable HL [19].

## 5. Radiotherapy Techniques and Novel Methods

**Figure 2:**
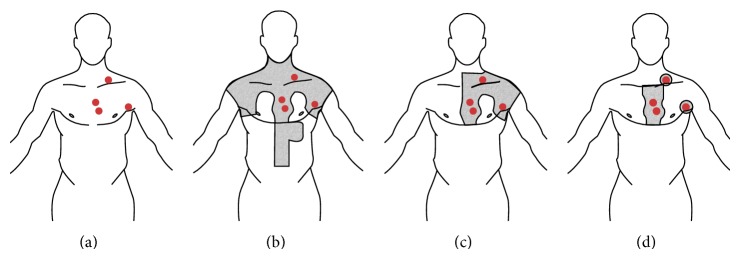
Images demonstrate changes in radiotherapy surface for HL. In this picture lymph nodes involved with HL disease are illustrated in red. In grey is depicted irradiated field region. (a) Involved lymph nodes, (b) mantle field, (c) IF-RT: involved field radiation therapy, and (d) IN-RT: involved nodal radiation therapy.

50 years ago RT was the only curative treatment strategy for patients with HL diagnosis. At the beginning use of RT was based on EF-RT, where not only involved site was irradiated but also lymphatic groups in the same region of the body. EF-RT was divided into mantle field (areas above the diaphragm) and para-aortic-splenic field (subdiaphragmatic field) also known as “invertic Y.” Mantle field covered the cervical, the mid-chest, and the axillary lymph nodes and the shape of the irradiated region looks like a type of cloak. A standard inverted Y field covers all para-aortic, iliacal, and inguinal lymph nodes as well as upper femoral nodes. In the most common disease localization in mediastinum and neck subtotal nodal irradiation (STNI) was standard procedure. STNI is concerned with huge region of the body including cervical, axillary, mediastinal, hilar, and para-aortic lymph nodes [39]. When the disease spread on both sides of diaphragm, total lymphoid irradiation (TLI), as a connection of mantle and inverted Y, was indicated. Moreover, used doses were huge with 44 Gy at Stanford, or even higher delivered to heart and breast. Older and currently used RT methods are depicted in Figure 2.

As a consequence both lower dose and limited fields were emerged to reduce the risk of RT-related toxicity. It was already proven that reduction of field size from extended to involved did not result in decreased efficacy of the treatment [40]. There were no statistically significant differences observed in CR compared EF-RT to IF-RT (98.5% and 97.2%), progression of the disease (0.8% and 1.9%), relapse (6.4% and 7.7%), and deaths (8.1% and 6.4%). What is more, according to HD8 trial of the GHSG, side effects are less frequent including leukopenia, thrombocytopenia, gastrointestinal toxicity, nausea, andloss of taste with no difference in late side effects (secondary neoplasia 4.5% and 2.8%, resp.) [40]. As a result, currently, IF-RT is considered to be the mainstay of care in early stage HL, delivered with three-dimensional conformal radiation therapy (3D-CRT). The standard volume at mean dose of 30 Gy, with a fractionation scheme of 2 Gy in 15 fractions.

That was the reason to further search novel, less harmful for healthy tissues, methods of RT. While IF-RT focus on lymph node region, where the disease was located during the diagnosis, involved nodal radiation therapy (IN-RT) is designed to eradicate lymph nodes that are enlarged after chemotherapy. This method, first developed by EORTC/GELA group, allows to protect normal tissues from radiation. This hypothesis was proven in small study, where the reduction of doses delivered with IN-RT compared to IF-RT resulted in a significant decrease in total body dose, particularly for 50% lower heart dose and 42% lower breast dose [41]. In larger study by Campbell et al. no increase in relapses was observed while EF-RT and IF-RT were compared to IN-RT [42]. There were 12 relapses observed in all investigated groups: four after EFRT (3%), five after IFRT (5%), and three after INRT (3%). Moreover there was no recurrence after IN-RT in lymph nodes with size less than 5 cm. For sure, in future randomized studies are necessary to introduce IN-RT as a standard method of treatment.

Nowadays, gold standard for external beam RT is 3D-CRT that use a linear accelerator (LINAC). In this case the tumor mass and normal tissues are delineated using accurately coregistered CT and MRI. Lately, while different RT techniques are under investigation, the breakthrough is intensity modulated radiation therapy (IM-RT). By IM-RT technique it becomes possible to modulate the intensity of every radiation beam, that as a result, influenced a highly precise dose of total radiation delivery. That was the main benefit when compared with 3D-CRT. According to study by Goodman et al. in HL patients, those who applied IM-RT had lowered the risk of pulmonary toxicity for approximately 14%. Moreover, heart and coronary protection was observed, when compared to standard 3D-CRT method [43]. Recently introduced radiotherapy planning and delivery techniques through reduced radiation volumes to healthy organs are intended to minimize dose-related effects, including heart and lung diseases, hypothyroidism, or secondary cancers [44]. Unfortunately, so far there are no data that are able to demonstrate a clinical benefit for replacing 3D-CRT with IM-RT in IF-RT.

Another novel RT technique being widely investigated in HL patients is deep-inspiration breath-hold radiotherapy (DIBH). Similar to IM-RT DIBH mainly focus on minimizing the dose of irradiation delivered to healthy organs mainly heart by increasing the distance between the heart and the irradiated region [45]. DIBH techniques may be introduced with intensity modulation techniques, such as volumetric arc therapy (VMAT). Currently, in a study by Paumier et al. it was discovered that radiation exposure of the coronary arteries, heart, and lungs in HL patients with mediastinal disease, was much decreased, while DIBH with IM-RT and/or VMAT techniques were used [46]. The most noticeable benefit was observed when the tumors were localized in the upper part of the mediastinum.

Recently it was reported that helical tomotherapy (TOMO) could reduce to dose of radiation on breasts, lung, heart, and thyroid gland in HL patients [47]. TOMO is able to deliver treatment that will not be possible by conventional RT methods, including multiple mediastinal lymph nodes irradiation. What is more this technique can be detected in high risk of radiation-induced toxicity patients, including those with acquired immunodeficiency [48] or treated with concurrent targeted medications [49]. This can be great solution for relapsed and refractory patients, who have already received multiagent chemotherapy. Moreover, TOMO might provide safer and more accurate RT profile for selected HL patients with bulky residual disease. Last but not least, according to preliminary results TOMO might be administered for total lymphoid irradiation as the preparation for allogeneic bone marrow transplantation or as an alternative therapy for chronic graft-versus-host disease [50].

Lately, huge interest is also applied to proton therapy that delivers a lower dose of irradiation to normal tissues compared to standard X-ray therapy. The main advantage of this therapy is the ability to localize the radiation dose more precisely and control where the proton releases the bulk of its cancer-fighting energy, although the exact dose generated from neutrons is still a concern [51]. Currently, a huge progress in comparison to second malignancies after photon and proton therapy as well as realistic calculations of stray radiation dose has been achieved [52, 53]. According to preliminary data, including high-dose treatments, proton therapy revealed very few organ toxicity; however this technique needs further randomized clinical trials [54].

## 6. Conclusions

Morbidity and mortality described among HL patients are mainly based on outdated RT treatment. Currently, modern RT methods deliver substantially less radiation to smaller body region than it was two or three decades ago. It is difficult to evaluate the risk of novel therapy including IF-RT or IN-RT when there is no long-term observation in data published so far. A great prospective for future could be development of more effective RT methods with reduced toxicity at the same time. Moreover, also predicting risk of recently introduced, sophisticated RT techniques such as TOMO or proton therapy is difficult due to lack of epidemiological data so far.
